# Quantification of pharyngeal airway space changes after two-jaw orthognathic surgery in skeletal class III patients

**DOI:** 10.1186/s12903-023-03075-y

**Published:** 2023-06-01

**Authors:** Ziqi Zhang, Shuze Wang, Jing Li, Zhijie Yang, Xia Zhang, Xiaofeng Bai

**Affiliations:** Department of Oral and Maxillofacial SurgerySchool and Hospital of StomatologyLiaoning Provincial Key Laboratory of Oral Diseases, China Medical University, Shenyang, 110002 China

**Keywords:** Orthognathic surgery, Pharyngeal airway space, Skeletal class III malocclusion; CT

## Abstract

**Background:**

Skeletal class III malocclusion is a common dentofacial deformity. Orthognathic treatment changes the position of the jaws and affects the shape of the upper airway to some extent. The aim of this study was to use multislice spiral computer tomography data and orthognathic knowledge to quantify the relationship between the amount of surgical movement of the maxilla or mandible in all three spatial planes and the changes in airway volume that occurred.

**Methods:**

A retrospective study of 50 patients was conducted. Preoperative and postoperative linear changes related to skeletal movements of the maxilla and mandible were measured and compared to changes in the most constricted axial level (MCA) and its anteroposterior (MCA-AP) and transverse diameters (MCA-TV). Correlation tests and linear regression analysis were performed.

**Results:**

Significant interactions were observed between the anterior vertical movement of the maxilla and the MCA-AP. The anteroposterior movement distance of the mandible was significantly correlated with changes in the oropharyngeal, velopharyngeal, total airway volume, MCA, MCA-AP, and MCA-TV. The change in the mandibular plane angle was significantly correlated with the change in velopharyngeal volume, total airway volume (nasopharynx, oropharynx, velopharynx), and MCA.

The linear regression model showed that oropharyngeal volume decreased by 350.04 mm^3^, velopharyngeal volume decreased by 311.50 mm^3^, total airway volume decreased by 790.46 mm^3^, MCA decreased by 10.96 mm^2^ and MCA-AP decreased by 0.73 mm^2^ when point B was setback by 1 mm.

**Conclusions:**

Anteroposterior mandibular control is the key to successful airway management in all patients. This study provides estimates of volume change per millimeter of setback to guide surgeons in treatment planning.

## Background

Skeletal class III malocclusion is a common dentofacial deformity. This type of malocclusion is abnormal development of the jaw caused by heredity, disease, and other congenital or acquired factors. Skeletal class III malocclusion, with its correlated facial appearance abnormalities, is often accompanied by low chewing efficiency, unclear pronunciation, and other functional disorders, and often leads to psychological problems in patients [1]. The literature reveals the high efficacy of orthognathic surgery in improving facial appearance, self-confidence, and quality of life [2]. The changes in the upper airway morphology after orthognathic surgery to correct jaw deformity have attracted great attention from clinicians in recent years. The upper airway is connected to the skull bones through soft tissues, such as muscles and ligaments [3]. The supra- and infra- hyoid muscles play an important role in the position of the mandible and maintenance of respiratory function [4]. Orthodontic-orthognathic treatment changes the position of the jaws and affects the shape of the upper airway to some extent. A previous report demonstrated that bimaxillary setback significantly decreased the volume and minimum axial area of the upper airway [5]. In addition, studies have shown that the minimum airway cross-sectional area is associated with the development of obstructive sleep apnea (OSA) [6, 7]. Therefore, the probability of severe OSA is high, when the cross-sectional airway area is less than 52 mm^2^. The probability is intermediate if the airway is 52–110 mm^2^, and low if the smallest airway area is more than 110 mm^2^ [8]. Research on the airway has aided oral surgeons in predicting the potential and realizing airway changes that occur during surgery.

It makes sense to visualize these airway changes during the diagnosis and treatment planning stages and quantification the amount of postoperative airway changes. Before the appearance of computed tomography (CT), lateral cephalometric radiographs have always been the main data for the study of the upper airway. The upper airway is a three-dimensional (3D) structure, while the cephalometric can only show the sagittal image of the upper airway. In contrast, 3D scans can provide accurate information on changes in the pharyngeal airway space (PAS) in terms of volume, depth, and length [9].

Owing to the large density difference between the upper airway and surrounding soft tissue in multislice spiral CT (MSCT) images, the upper airway boundary can be directly and clearly seen, which improves the accuracy of upper airway sectioning [10, 11]. At the same time, it can directly measure and analyze the cross-sectional shape, cross-sectional area, and volume of the upper airway, and make the measurement of the upper airway more comprehensive. Therefore, this study chose MSCT instead of cone beam CT (CBCT) for this experiment.

In recent years, the number of 3D studies on upper airway morphology have increased gradually. Most of the studies have focused on the effect of simple mandibular setback on PAS or the comparison of the effect of one-jaw and two-jaw surgery on PAS, and there have been fewer studies on the influence of different directions of maxillary movements on the airway. In a meta-analysis, He et al. [12] compared the effects of isolated mandibular setback and bimaxillary surgery and reported that isolated mandibular setback reduced upper airway volume, but bimaxillary surgery did not result in any significant reduction. Studies have shown that simple mandibular setback contracts the oropharyngeal, velopharyngeal, and total airway and is maintained for a long time [13]. In patients undergoing 2-jaw surgery with mandibular setback and maxillary advancement, airway volume loss is reduced, and increased airway volume in some areas improves overall airway loss [14]. Therefore, the question of whether bimaxillary surgery can affect PAS is controversial, and for this study we selected only patients with skeletal class III diagnosis and two-jaw surgery.

The research is mostly limited to the direction of changes in upper airway morphology. There are a few pieces of literature that quantify the linear, area, and volumetric changes per millimeter (mm) advancement or setback of jaws. Hart et al. reported the quantitative effects of anteroposterior and vertical maxillary movements on the airways [15]. However, the effects of the occlusal plane angle and mandibular plane angle on PAS have not been described. This work studied the occlusal plane and mandibular plane angles based on skeletal movements.

Therefore, the aim of this study was to quantify the relationship between the amount of surgical movement of the maxilla or mandible in all three spatial planes and the changes in airway volume that occurred based on MSCT data and knowledge of orthognathics.

## Methods

We designed a retrospective study to assess the airway changes. All patients who attended the Hospital of Stomatology of China Medical University from January 2018 to December 2020 and met the inclusion criteria were included. The study sample comprised a total of 50 patients, 26 males (52%) and 24 females (48%), with an average age of 23.92 ± 4.39 (range 19 to 38). Acquisitions took place at a mean of 3.2 weeks before surgery (T0), and 10.6 months after surgery (T1). All patients received presurgical and postsurgical orthodontic treatment, received treatment with their own or guardian's consent and signed the consent form. The following inclusion criteria were used: (a) All subjects were diagnosed with skeletal class III deformity and a mesial molar relationship. (b) The patients were selected based on the extent of the menton (Me) deviation from the midsagittal reference plane [16], with a Me deviation of less than 2 mm. (c) Body mass index (BMI) 18.5–23.9 kg/m^2^,circumference < 43 cm (17 inch) for males, and for female < 40 cm (16 inch) were selected [17]. The following exclusion criteria were used: (a) Patients with excessive BMI changes before and after surgery. (b) Patients with a maxillary deficiency or the presence of a syndrome or cleft lip and palate. (c) Patients with symptoms of a disorder or degeneration of the temporomandibular joint, and those who had undergone maxillofacial surgical procedures or received treatment for the correction of sleep apnea. All surgical procedures were completed by the same oral surgeon who used the same surgical and fixation plan (LeFort I osteotomy and bilateral sagittal mandibular ramus osteotomy and rigid internal fixation) for each patient. All the patients were simulated before operation using CAD, and the surgical planning were transferred to operations by 3D printing templates. This retrospective study was approved by the institutional ethics committee. The study design fulfils the guidelines of the Declaration of Helsinki regarding ethical principles for medical research involving human subjects.

MSCT scanning was required for all patients preoperatively (T0) and postoperatively (T1). All MSCT data of the study subjects were obtained using the same TOSHIBA X-ray high voltage (Aquilion TSX-101A) machine with a voxel size of 0.3 mm. All scans were performed at 50 mA at 120 kV, and the scans were reconstructed at 0.3 mm and exported in DICOM format. All MSCTs were completed by a single photographer. To minimize distortions and obtain optimal MSCT quality, the patient was in a supine position during the scanning, with the occlusal position in the intercuspal position. The patient held his breath at the end of breath without swallowing and kept strictly motionless during the scanning [18]. The upper and lower lips are closed naturally, while the muscles of the tongue and mouth remain relaxed. The data were imported into the Mimics 15.0 (Materialise, Inc., Belgium) software. Maxillary and mandibular alveolar bone around teeth and separated teeth were digitally removed from the whole image by threshold segmentation according to the difference in X-ray transparency. Park et al. [14] used a range of -1024 to -600 Hounsfield units for airway volume measurements using CBCT. However, in the MSCT, the threshold may vary. When the threshold is set at a range of -1024 HU to -328 HU, the pharyngeal airway can be effectively distinguished from the adjacent soft tissues. 3D reconstruction of the upper airway was performed. All MSCT analyses were performed by 1 examiner.

**Fig. 1:**
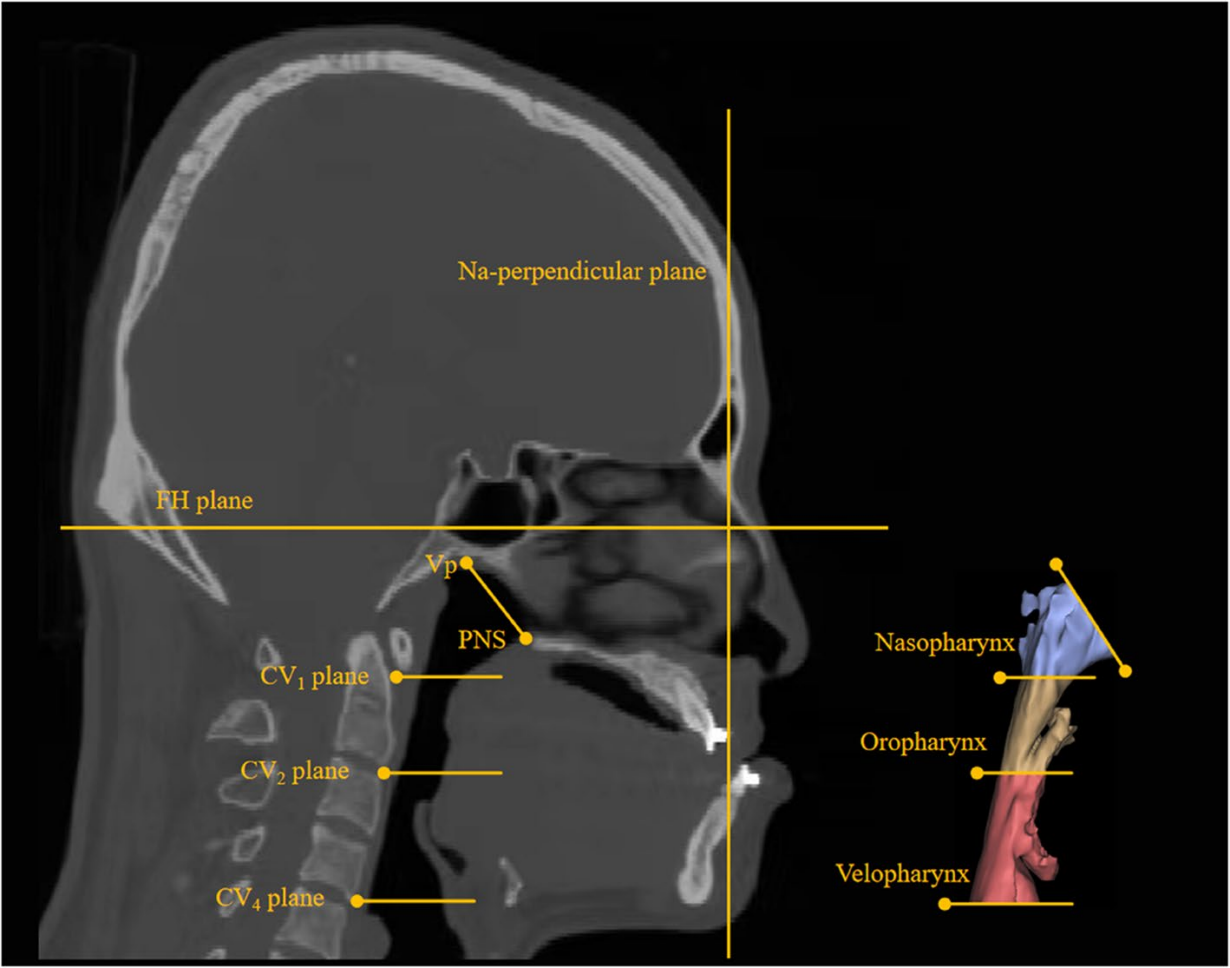
Partition of the upper airway. The airway levels are parallel to the Frankfort horizontal (FH) plane. The CV1, CV2, and CV4 planes are parallel to the FH plane through the most inferior point of each corresponding cervical vertebra. Na-perpendicular plane is the midsagittal plane passing through Na and perpendicular to the FH. PNS: Posterior nasal spine; Vp: The most posterior part of the ala of vomer; Nasopharynx: Region between PNS-Vp and CV1; Oropharynx: Region between CV1 and CV2; Velopharynx: Region between CV2 and CV4

The upper airway zone was analyzed using Park's method [14]. The images were adjusted so that the Frankfort anteroposterior (FH) plane (the plane constructed on both sides of Porion and both sides of Orbitale) was parallel to the anteroposterior plane. The midsagittal plane perpendicular to the FH plane was determined to be passing through the most anterior point of the frontonasal suture in the midsagittal plane, Nasion (Na) and the most posterior inferior point of the occipital bone at the anterior margin of the foramen magnum, Basion (Ba). The most anterior and inferior points of the first, second, third, and fourth cervical vertebrae were used to make a plane parallel to the FH plane. They were recorded as the first cervical vertebra plane (CV1 plane), the second cervical vertebra plane (CV2 plane), the third cervical vertebra plane (CV3 plane), and the fourth cervical vertebra plane (CV4 plane). PNS-Vp plane was made through the posterior nasal spine (PNS)and the most posterior point of the ala of the vomer (Vp). The upper airway was divided into three segments: ① the nasopharyngeal airway was located between the CV1 plane and the posterior nasal spine-piriform plane; ② the oropharyngeal airway was located between the CV1 and CV2 planes; and ③ the velopharyngeal airway was located between the CV2 and CV4 planes (Fig. 1).

The anteroposterior and vertical positions of the deepest anterior point in the concavity of the anterior maxilla (point A), the deepest anterior point in the concavity of the anterior mandible (point B), and PNS were recorded using linear measurements perpendicular to the FH plane and the midsagittal plane. Distance changes were recorded in mm. Upward and forward skeletal movements were positive, and downward and backward skeletal movements were negative. Postoperative changes of airway were compared to skeletal movements of the maxilla and mandible (position of points A, B, PNS, mandibular plane angle, and occlusal plane angle).

**Fig. 2:**
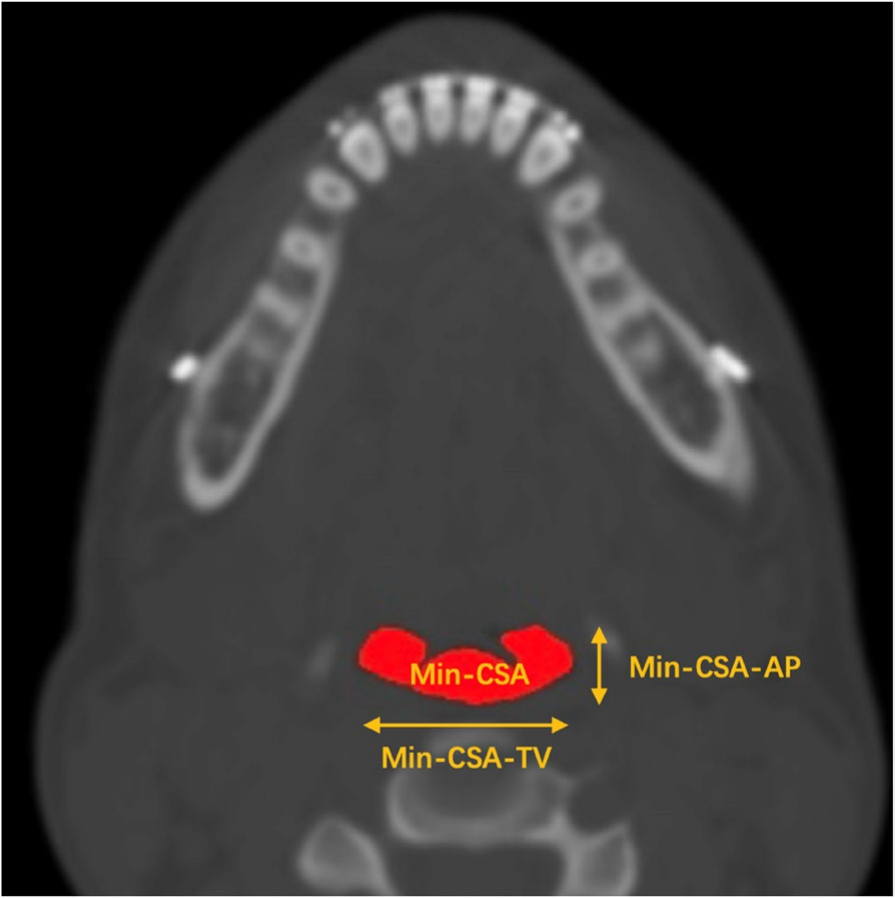
Minimum cross-sectional area measurements. MCA: Minimum cross-sectional area; MCA-AP: Anteroposterior diameters of MCA; MCA-TV: Transverse diameters of MCA

The most constricted axial level of the total pharyngeal airway was defined as the minimum cross-sectional area (MCA). The anteroposterior (MCA-AP) and transverse (MCA-TV) diameters of MCA were measured (Fig. 2) [19]. The area changes were recorded in mm^2^. Compared to changes in the MCA, MCA-AP, and MCA-TV preoperative and postoperative. The total airway pharynx was defined as the sum of the nasopharynx, oropharynx, and velopharynx. Changes in the nasopharyngeal, oropharyngeal, velopharyngeal, and total volume of the upper airway were measured. Volume measurements were recorded in mm^3^. A positive value indicates increased airway volume and a negative value indicates reduced airway volume.

The measured values were imported into SPSS Statistics, version 24.0 (IBM Corp., Armonk, NY, USA) for data analysis, and the Kolmogorov Smirnov test was performed to determine whether values were normally distributed. Accordingly, the means of skeletal movements and airway changes at T0 and T1 were compared using a paired t-test. Pearson correlation or Spearman correlation tests were performed on all subjects to analyze the relationship between linear measurement changes in skeletal position and changes in airway morphology. Multiple linear regression analysis was used to evaluate the relationship between linear measurement changes and airway morphology changes, and a linear regression equation was obtained. Statistical significance was set at *P* < 0.05.

## Results

A comparison of cephalometric measurements is shown in Table 1. The changes in the airway are represented absolute volume (mm3), area (mm2), or linear distance (mm) (Table 2). Significant changes were observed between the preoperative and postoperative vertical movements at point A (*P* = 0.028), anteroposterior movements of point A (*P* = 0.014), vertical movements of point B (*P* = 0.000), vertical movements of point PNS (*P* = 0.001), and anteroposterior movements of point PNS (*P* = 0.013). No significant changes were seen in volumetric measurements, except for significant differences between preoperative and postoperative MCA (*P* = 0.005), MCA-TV (*P* = 0.010), and MCA-AP (*P* = 0.014).

**Table 1:** The comparisons of skeletal linear measurements before operation (T0) and after operation (T1)

	Movement directions	T0	T1	T1-T0	*P-value*
Point A (mm)	Anteroposterior	0.66(3.88)	1.59(3.53)	0.93(2.54)	0.014*
	Vertical	32.66(3.33)	33.36(3.75)	0.70(2.19)	0.028*
Point B (mm)	Anteroposterior	4.71(6.72)	0.99(6.02)	-3.72(4.16)	0.000**
	Vertical	70.85(10.16)	69.28(9.17)	-1.57(10.50)	0.296
Point PNS (mm)	Anteroposterior	41.59(4.76)	40.06(4.41)	-1.53(4.20)	0.013*
	Vertical	24.65(2.56)	23.73(2.82)	-0.92(1.79)	0.001**
Mandibular plane Angle (°)		24.78(6.25)	25.04(5.94)	0.26(3.69)	0.626
Occlusal plane Angle (°)		6.10(3.45)	6.59(3.52)	0.49(2.89)	0.236

All data presented as mean (SD)

*P-value*: * < 0.05, ** < 0.01

**Table 2:** The changes of the volumetric, area, and linear parameters before operation (T0) and after operation (T1)

Airway changes	T0	T1	T1-T0	P
Volume				
Nasopharynx(mm^3^)	9484.23(2719.56)	9658.57(2890.94)	174.34(1983.20)	0.537
Oropharynx(mm^3^)	7762.84(3146.24)	7275.49(3578.92)	-487.36(2446.79)	0.165
Hypopharynx(mm^3^)	10,509.93(3596.91)	9848.32(3822.54)	-661.61(2341.71)	0.051
Total pharynx(mm^3^)	27,757.01(7950.94)	26,782.39(1271.56)	-974.63(5372.76)	0.206
Area				
MCA(mm^2^)	244.32(110.88)	210.38(105.06)	-33.94(82.37)	0.005**
Liner				
MCA-TV(mm)	24.24(7.51)	23.88(6.92)	-0.36(5.99)	0.676
MCA-AP(mm)	12.87(4.36)	11.81(3.64)	-1.06(2.94)	0.014*

All data presented as mean (SD)

*P-value*: * < 0.05, ** < 0.01

Significant interactions were observed between anterior vertical movement of the maxilla and MCA-AP (*P* = 0.010). The anteroposterior movement distance of the mandible is significantly correlated with the change of oropharyngeal (*P* = 0.000), velopharyngeal (*P* = 0.000), total airway volume (*P* = 0.000), MCA (*P* = 0.000), MCA-AP (*P* = 0.0001) and MCA-TV (*P* = 0.015). The change in the mandibular plane angle was significantly correlated with the change in velopharyngeal volume (*P* = 0.017), total airway volume (*P* = 0.046), and MCA (*P* = 0.030). No significant differences in nasopharyngeal, oropharyngeal, velopharyngeal, and total volume of the upper airway between the preoperative and postoperative periods were observed in this study (Table 3). In addition, no significant effect of skeletal movements on airway changes was observed, except for anterior vertical movement of the maxilla, anteroposterior movement distance of the mandible, and the change in mandibular plane angle. This is possibly because all samples were considered as a whole, and the direction of maxillary movement was not distinguished. This work divided maxillary movements into maxillary advancement and maxillary setback and conducted correlation tests. The test results are shown in Table 4, and the anteroposterior movements of point A still had no significant effect on PAS.

**Table 3:** The relationship between skeletal movements and changes of upper airway

		Nasopharyngeal airway change(mm^3^)		Oropharyngeal airway change(mm^3^)		Hypopharyngeal airway change(mm^3^)		Total airway change(mm^3^)		MCA (mm^2^)		MCA-TV (mm)		MCA-AP (mm)	
	Movement directions	rho	*P*	rho	*P*	rho	*P*	rho	*P*	rho	*P*	rho	*P*	rho	*P*
Point A (mm)	Anteroposterior	-.002	0.991	.076	0.599	-.095	0.512	-.066	0.650	-.093	0.521	-.076	0.602	.026	0.856
	Vertical	-.011	0.938	-.272	0.056	-.132	0.362	-.192	0.181	-.259	0.069	-.145	0.316	-.361*	0.010
Point B (mm)	Anteroposterior	.180	0.212	.546^**^	0.000	.562^**^	0.000	.556^**^	0.000	.560^**^	0.000	.473^**^	0.001	.344^*^	0.015
	Vertical	.087	0.548	-.026	0.860	-.157	0.277	-.060	0.678	-.152	0.293	-.088	0.544	.065	0.653
Point PNS (mm)	Anteroposterior	-.226	0.114	-.206	0.151	-.031	0.829	-.210	0.143	-.151	0.294	-.110	0.448	-.232	0.105
	Vertical	.117	0.420	-.107	0.461	-.077	0.596	-.003	0.983	.098	0.499	.205	0.153	.084	0.560
Mandibular plane Angle (°)		.015	0.916	-.262	0.066	-.336^*^	0.017	-.348^*^	0.046	-.308^*^	0.030	-.215	0.133	-.205	0.153
Occlusal plane Angle (°)		-.066	0.650	.030	0.835	-.067	0.645	.004	0.978	-.131	0.365	-.150	0.299	-.049	0.736

*P-value*: * < 0.05, ** < 0.01

rho: Pearson’s or Spearman’s correlation coefficient

**Table 4:** The relationship between maxilla movements and changes of upper airway

		Nasopharyngeal airway change		Oropharyngeal airway change		Hypopharyngeal airway change		Total airway change		MCA (mm^2^)		MCA-TV (mm)		MCA-AP (mm)	
	Movement directions	rho	*P*	rho	*P*	rho	*P*	rho	*P*	rho	*P*	rho	*P*	rho	*P*
Point A (mm)	Advancement	.017	0.932	-.276	0.148	-.107	0.581	.061	0.753	-.198	0.303	-.155	0.422	.081	0.675
	Setback	.329	0.145	.234	0.306	.253	0.268	.068	0.771	.141	0.541	-.073	0.754	.094	0.686

*P-value*: * < 0.05, ** < 0.01

rho: Spearman’s correlation coefficient

**Fig. 3:**
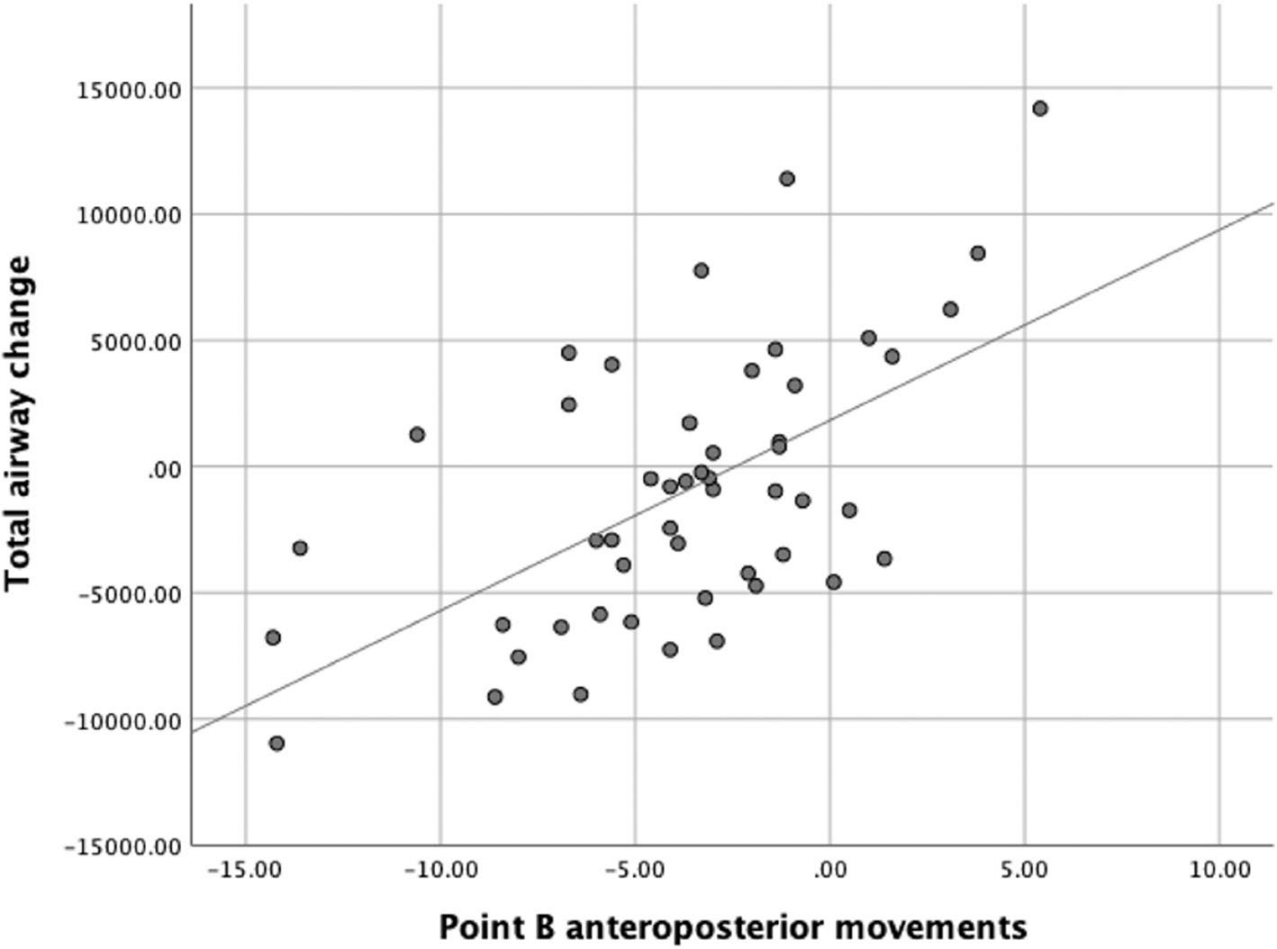
Linear regression of Point B anteroposterior movements and Total airway change

Linear regression analyses quantifying the association among individual linear measurements, airway volume, and area changes are presented in Table 5. The linear regression model showed that oropharyngeal volume decreased by 350.04 mm^3^(*P* = 0.000), velopharyngeal volume decreased by 311.50 mm^3^(*P* = 0.001), total airway volume decreased by 790.46 mm^3^(*P* = 0.000) (Fig. 3), MCA decreased by 10.96 mm^2^ (*P* = 0.002), and MCA-AP decreased by 0.73 mm^2^ (*P* = 0.008) when point B was setback by 1 mm. However, the vertical movements of point B (*P* < 0.05) had no significant influence on airway morphology. There was also no significant effect of point A (*P* > 0.05) and point PNS (*P* > 0.05) on anteroposterior or vertical movements of the upper airway.

**Table 5:** The Influence of surgical skeletal movements on changes in airway space

		Nasopharyngeal airway change		oropharynx airway change		velopharynx airway change		Total airway change		MCA(mm^2^)		MCA-TV(mm)		MCA-AP(mm)	
	Movement directions	β	*P*	β	*P*	β	*P*	β	*P*	β	*P*	β	*P*	β	*P*
Point A (mm)	Anteroposterior	-9.550	0.945	-42.772	0.762	-184.273	0.194	-236.594	0.461	-4.948	0.340	-0.057	-0.286	-0.251	0.537
	Vertical	27.969	0.858	-127.324	0.423	163.898	0.301	64.544	0.857	-2.264	0.696	-0.395	-1.767	0.044	0.923
Point B (mm)	Anteroposterior	134.917	0.139	350.044**	0.000	311.498**	0.001	796.459**	0.000	10.958**	0.002	0.185	1.432	0.727**	0.008
	Vertical	19.695	0.541	-24.109	0.460	0.244	0.994	-4.170	0.955	-0.847	0.478	-0.020	-0.425	-0.042	0.654
Point PNS (mm)	Anteroposterior	-134.444	0.110	-110.227	0.193	-10.178	0.903	-254.849	0.185	-2.414	0.433	-0.151	-1.274	-0.077	0.750
	Vertical	176.200	0.362	-171.791	0.380	-322.541	0.102	-318.13	0.473	1.833	0.797	0.020	0.740	0.319	0.570
Mandibular plane Angle (°)		72.699	0.581	-71.900	0.590	-147.509	0.270	-146.709	0.628	0.008	0.999	0.022	0.116	0.165	0.667
Occlusal plane Angle (°)		139.075	0.222	211.153	0.07	-10.587	0.926	339.640	0.195	0.570	0.892	0.088	0.546	-0.067	0.838

*P-value*: * < 0.05, ** < 0.01

β: Linear regression coefficient

## Discussion

This study evaluated the effects of two-jaw orthognathic surgeries for Class III malocclusions on PAS. Furthermore, we developed an easy mathematical approach for predicting changes in the airway space after surgery based on MSCT.

When developing a statistical model that is capable of predicting surgical outcomes, possible sources of error should be eliminated. When comparing the standard deviations of landmark identification of the CBCTs and MSCT, it is striking that landmark identification in the later tended to be somewhat more precise [11]. All measurements of nasopharyngeal volume are reported to be less reliable than oropharyngeal volume because the nasopharyngeal region is more difficult to define, with the turbinate and concha regions constituting a complex anatomical structure [20]. To improve the reliability of measurements, we utilized skeletal markers and defined the nasopharynx restrictively in terms of the PNS-Vp and CV1 planes of the nasopharyngeal region [14]. These measurement targets included the nasopharynx, oropharynx, and velopharynx, with a wider range. Lin He et al. concluded that mandibular setback narrowed the CV3 and CV4 planes of the airway, and MCA was located between CV3 and CV4 in some cases [21]. Therefore, definition in this work of the velopharynx included the area between CV3 and CV4.

Orthognathic patients who underwent Le Fort I maxillary osteotomy and sagittal mandibular ramus cleavage were selected as the study subjects strictly according to the inclusion and exclusion criteria. The reduced airway dimensions immediately after surgery may be a result of postoperative soft tissue edema and inflammation, with the pharyngeal airway subsequently stabilized [17]. Postsurgical MSCT images were taken for a minimum of 4 months postoperatively to allow for the reduction of tissue inflammation [15]. Muscle adaptation occurs during the first 6 months after surgery [22]. In this study, the average duration from T0 to T1 was 10.6 months, which should allow enough time for muscle adaptation and the elimination of inflammation and edema. Fixed points and measurements were performed by the same person. To reduce the error, repeated measurements were carried out once a week, repeated three times, and the average value of the three measurements was taken. During MSCT, the patient was resting and quiet without local muscle movement or other external interference factors. Tissue structures can easily be changed by swallowing or respiratory movements during the tomography scan, and the MSCT examination is performed in a similar respiratory condition [23]. The most appropriate relationship between the pharyngeal airway morphology that was scanned in the supine position and apnea events at the same position.

It is widely accepted that the upper airway is affected by orthognathic surgery. Results of this work confirmed that the anteroposterior movement distance of the mandible was significantly correlated with changes in the oropharyngeal, velopharyngeal, total airway volume, MCA, MCA-AP, and MCA-TV, and that anterior vertical movement distance of the maxilla were significantly correlated with MCA-AP.

Some authors document that MCA is more relevant in pharyngeal airway studies than in volumetric analyses, because the degree of contraction is the most important factor of airflow resistance according to Poiseuille’s law [24]. The lumen size, collapsibility, airflow velocity, and turbulence are considered major determinants of airway patency [25]. A previous study showed that single-jaw surgery resulted in a reduction in MCA, but bimaxillary surgery did not affect MCA [14]. Another study reported that bimaxillary surgery for mandibular protrusion reduced MCA and MCA-AP [26]. Park et al. [27] reported a single mandibular setback that reduced MCA-AP but increased MCA-TV. Narrowing of the airway after mandibular setback surgery may increase the airflow rate and intraluminal pressure [28].

For every millimeter that a point is moved, there is a corresponding increase or decrease in the volume or area. For example, if an airway area gains 100 mm^3^ or 10 mm^2^ for every 1 mm forward movement of one point, then the airway area will also lose 100 mm^3^ or 10 mm^2^ for every 1 mm backward movement of the said point [15]. This principle can be applied to all anteroposterior and vertical movements of all points. When looking at the sample as a whole, regardless of the vertical movements of the mandible, this study found that the anteroposterior movement distance of the mandible was significantly correlated with the changes of oropharyngeal, velopharyngeal, total airway volume, MCA, and MCA-AP. The linear regression model showed that for every mm of mandibular setback, 790.46 mm^3^ of total airway volume, 350.04 mm^3^ of oropharyngeal volume, and 311.50 mm^3^ of velopharyngeal volume were lost. This movement negatively affected MCA by 10.96 mm^2^ and MCA-AP by 0.73 mm. The advancement movements of mandibular increases airway for the same values. This makes sense given the number of soft tissue attachments and the close relationship between the mandible and the airway support structures. This movement affects the most constricted areas; MCA decreased by 10.96 mm^2^ and MCA-AP decreased by 0.73 mm^2^ when mandibular was setback by 1 mm. This needs to be noted because it may have clinical significance when considering the inverse relationship between the most constricted area and flow resistance. These findings support the negative effects of orthognathic surgery on the airways of class III patients, and provide predictability of volume change per mm of setback to guide surgeons in treatment planning.

The change in the mandibular plane angle was significantly correlated with the change in velopharyngeal volume, total airway volume, and MCA. This may be because the change in the mandibular plane angle affects the position of the hyoid bone and tongue and indirectly changes the airway shape. No significant influences were observed between other linear measurements and changes in airway space, except for the anteroposterior movement distance of the mandible and the change in mandibular plane angle. During orthognathic operation, the mandible of a skeletal class III malocclusion patient setbacks in a short period, resulting in a significant decrease in the volume of the upper airway. Maxillary movement also affects airway volume [26].

In this study, the position change of the posterior maxilla (point PNS) did not affect airway morphology, and changes in the position of the anterior maxilla (point A) also had no effect on airway morphology. This may be because maxillary movements are not significant surgical movements and result in minimal changes in the airway when the sample is taken as a whole. We grouped the cases of maxillary advancement and maxillary setback into groups and performed correlation tests separately and found no significant effect of anteroposterior movement of the maxilla on the upper airway. This may be due to the excessive dispersion of maxillary motion data or measurement errors.

These findings suggest that anteroposterior mandibular control is key to successful airway management in all patients and supports many of the claims made in earlier studies about the negative effects of mandibular setback on total airway volume in class III patients. The results also provide estimates of volume change per mm of setback to guide surgeons in treatment planning. These findings are also in agreement with the published information on the pretreatment volumetric values of different skeletal malocclusions and the types of surgical movements needed for their correction.

No clinical data are expressed regarding the improvement of airway and breathing functioning of the patients in this text. Some kinds of polysomnographic or airway flow measurements will be added in the future to objectify the clinical impact of decreasing the upper airway following bimax surgery. And we need to refine the influence of age, gender, race, region, BMI, lifestyle, breathing style and other factors on upper airway morphology.

## Conclusions

According to the results of this study, mandibular setback significantly reduced airway volume and MCA, the greater the distance, the more significant was the airway reduction. The mathematical approach used in this work seemed to predict the development of PAS by measuring the patients’ preoperative imaging. A good treatment plan reduces the incidence of medically induced sleep apnea syndrome while ensuring the treatment effect, by reducing the magnitude of narrowing of the airway and avoiding excessive mandibular constriction.

## Data Availability

The datasets generated and/or analysed during the current study are not publicly available due follow-up research but are available from the corresponding author on reasonable request.

## Abbreviations

Na: The most anterior point of the frontonasal suture in the midsagittal plane
Ba: The most posterior inferior point of the occipital bone at the anterior margin
Or: The most inferior point of the orbital margin
Po: The most superior point of the external auditory meatus
A-point: The deepest anterior point in the concavity of the anterior maxilla.
B-point: The deepest anterior point in the concavity of the anterior mandible
PNS: The most posterior point of the hard palate
Me: The most inferior mid point of the chin on the outline of the mandibular symphysis
Vp: The most posterior point of the ala of the vomer
FH: The plane was constructed on both side of Po and right of Or
Midsagittal: The plane was perpendicular to the FH plane passing through Na and Ba
Na-perpendicular: The plane was perpendicular to the FH and the midsagittal planes passing through Na
CV1 plane: The plane was parallel to the FH plane passing through CV1
CV2 plane: The plane was parallel to the FH plane passing through CV2
CV3 plane: The plane was parallel to the FH plane passing through CV3
CV4 plane: The plane was parallel to the FH plane passing through CV4
PNS-Vp plane: The plane was perpendicular to the midsagittal plane passing through PNS and Vp
DICOM: Digital imaging and communication in medicine
Mimics: Materialise's interactive medical image control system
STL: Standard Tessellation Language
OSA: Obstructive Sleep Apnea
CBCT: Cone Beam Computer Tomography
MSCT: Multislice spiral CT
BSSRO: Sagittal split ramus ostcotomy
PAS: Pharynx airway space
MCA: The minimum cross-sectional area of the upper airway
MCA-TV: The transverse line on the greatest transverse dimension at MCA
MCA-AP: The sagittal line on the greatest sagittal dimension at MCA
